# The usefulness of measuring the anion gap in diagnosing metformin-associated lactic acidosis: a case series

**DOI:** 10.1186/s13256-020-02655-8

**Published:** 2021-01-21

**Authors:** Jesus Ruiz-Ramos, Laura Lozano-Polo, Ana Juanes-Borrego, Iván Agra-Montava, Mireia Puig-Campmany, María Antonia Mangues-Bafalluy

**Affiliations:** 1 Pharmacy Department, Hospital Santa Creu i Sant Pau, C/San Quintín 89, 08041 Barcelona, Spain; 2 Emergency Department, Hospital Santa Creu i Sant Pau, Barcelona, Spain

**Keywords:** Metformin, Acidosis, Acute renal failure, Emergency care, Diabetes, Anion gap

## Abstract

**Background:**

Metformin-associated lactic acidosis (MALA) is a widely documented adverse event of metformin. Despite being considered one of the main causes of metabolic acidosis, the association between an anion gap and MALA diagnosis is still uncertain.

**Case presentation:**

Cases involving six Caucasian patients with suspected MALA who were admitted to the emergency department were analysed. All these patients presented with pH values < 7.35, lactate levels > 2 mmol/L, and estimated glomerular filtration < 30 mL/min. Metformin plasma concentrations were > 2.5 mg/L in all the patients. The highest metformin concentrations were not found in the patients with the highest lactate levels. The anion gap values ranged from 12.3 to 39.3, with only two patients exhibiting values > 14.

**Conclusions:**

In patients with MALA, there is a significant variability in the anion gap values, which is not related to the level of metformin accumulation, and therefore, it is doubtful whether measuring anion gaps is useful as an approach for MALA diagnosis.

## Background

Since the 1950s, metformin has been successfully used as a first-line pharmacotherapy for treating individuals with type II diabetes [1]. Although it is considered as a drug with a broad safety profile, metformin-associated lactic acidosis (MALA) is a widely documented adverse event [2, 3]. MALA is a rare condition associated with poor prognoses and has an estimated mortality of between 25 and 50% [4]. It develops more frequently in elderly patients with impaired kidney function and liver failure [5]

It has been established that a pH bellow 7.35, a high lactate level, and compatible symptomatology are key elements in detecting the presence of MALA [5]. The anion gap, which measure the difference between cations and anions in serum, is an useful tool when attempting to identify the cause of metabolic acidosis. Despite a high anion gap being considered one of the main causes of metabolic acidosis, it is still uncertain whether there is an association between an anion gap and MALA diagnosis. In this report, we present a case series of suspected MALA patients with the aim of highlighting the importance of the anion gap value in MALA diagnoses.

## Case presentation

Cases involving six Caucasian patients with suspected MALA who visited the emergency department between September 2019 and December 2019 were analysed. The main characteristics of these patients are presented in Table 1. The mean age was 75.5 (standard deviation = 6.3) years. All the patients exhibited respiratory symptoms at admission; further, based on a blood gas analysis performed at emergency department admission, almost all of them presented with a pH value < 7.35 and lactate level > 2 mmol/L while only one patient had a lactate level of > 5 mmol/L, which is one of the established criteria for an MALA diagnosis [7]. Lactate level was analysed by the “GEM premier 3500 in Instrumentation Laboratory” automated blood gas analysis system blood gas analyser (ABL90, Radiometer, Bronshoj, Denmark). Alternative causes of metabolic acidosis such concomitant drugs or toxics substances were excluded, based on medical history review and patients or relatives interview. Four patients presented with chronic kidney disease as a comorbidity. However, the estimated glomerular filtration rate (calculated by the modification of diet in renal disease (MDRD) formula [6]) was less than 30 mL/min at admission in all cases. All the patients had metformin plasma concentrations above the upper limit of the therapeutic range (> 2.5 mg/L) [7]. The highest metformin concentrations were not found in patients with the highest lactate values.

**Table 1:** Characteristics of patients included

No	Age	CKD	Creatinine (mg/dL)	Metformin (mg/L)	pH	Lactate (mmol/L)	0 hour Anion gap	24 hours Anion gap	Exitus 24 hours
1	73	No	7.87	34.7	7.07	12.5	37.7	36.6	Si
2	77	Yes	2.02	10.32	7.03	2.7	16.7	17.1	No
3	82	Yes	4.14	44.66	7.13	2.8	12.3	14.2	Si
4	64	No	7.85	15.8	6.74	7.9	39.3	44.8	No
5	78	No	1.86	18.1	7.30	2	14.2	12.2	No
6	79	Yes	4.68	8.27	7.21	5.7	16.3	24.9	No

*CKD* chronic kidney disease

Only two of the included patients met the haemodialysis criteria established by the extracorporeal treatments in poisoning (EXTRIP) recommendations [8] (lactate > 20 mmol/L, pH ≤ 7.0, or failure of standard treatment, including bicarbonate); further, only one of them received haemodialysis, and therefore, the therapeutic effort upon admission was limited to the other patient. Two patients died during hospitalisation, and these patients had the highest metformin plasma concentrations (> 30 mg/L). The anion gap values (calculated as [Na^+^] + [K^+^] − [Cl^−^] − [HCO_3_^−^]) ranged from 12.3 to 39.3, with only two patients exhibiting values greater than 14.

## Discussion and conclusions

The results of our series demonstrate that in the case of severe metformin intoxication, the anion gap value may be normal or slightly elevated, with no relation to the metformin plasma concentrations. Anion gap alterations can occur in different situations involving acid-base imbalance, and monitoring these alterations is a useful approach in determining the aetiology of metabolic acidosis. One of the most common causes is lactic acidosis. Metformin inhibits cellular mitochondrial respiration, causing an increase in anaerobic metabolism and lactate production [9]. This situation leads to type B lactic acidosis [10] that overlaps with type A lactic acidosis due to hypoperfusion in patients with haemodynamic failure in case of systemic collapse.

The association between severity and mortality on admission and the lactate value is well known. However, the association between the anion gap value and mortality remains unclear. The sensitivity of using the anion gap has been reported to be poor in detecting the levels of acidosis when it is mediated by lactate [11]. In our patients, we observed that very high concentrations of metformin with an increase in lactate levels did not lead to a relevant increase in the anion gap. Therefore, this series reinforces the view that measuring the anion gap has limited utility in predicting the accumulation of this drug because of the different factors that can coexist in these patients. The toxicity found in our patients was possibly due to mixed acidosis caused by a combination of accumulated metformin, diabetic ketoacidosis, tissue hypoxia, and kidney failure, which resulted in a wide range of anion gap values. The absence of plasma albumin levels has prevented from obtaining corrected anion gap values, which is a limitation of our study since it could partly affect the results obtained. Among the limitations associated with our series, the one worth noting is that at admission, there was a lack of data on albumin levels that can significantly modify the value of the anion gap.

On the other hand, it is known that the severity of lactic acidosis is not proportional to metformin plasma concentrations, as patients described as having severe clinical symptoms exhibited slightly elevated concentrations, and those without MALA had high concentrations of metformin; despite these findings, high concentrations have been associated with higher mortality rates in patients with MALA [12]. The small sample size in our series prevented us from drawing conclusions regarding the association between metformin concentrations and mortality. Despite this, we observed that the patients with the highest metformin concentrations were those who died, which contributes to fuelling this debate.

Likewise, changes in pH, lactate levels, and bicarbonate values are critical for making a selection among therapeutic options. It should be noted that only one of the six patients had a lactate value of > 5 mmol/L, an established criterion for the diagnosis of MALA [5]. However, all the patients in our series had serious clinical conditions. For this reason, it must be considered that lower levels of lactate may indicate significant accumulations of metformin and severe clinical presentations, given that the anion gap value could not aid in diagnosing these cases.

Two of the six patients (33.3%) died during hospitalisation. Previous studies have reported mortality rates between 25 and 50% [4], highlighting the severity of this complication. Despite the fact that owing to its benefits in terms of the incidence of cardiovascular events and mortality, metformin is recommended by different guidelines for patients with mild or moderate impairment of renal function with an adequate dose adjustment [1], elderly patients often exhibit a sudden deterioration of renal function secondary to different circumstances. Our patients presented with very high metformin concentrations, which probably indicate renal function deterioration that evolved for several days before hospitalisation. The high distribution volume of metformin facilitates its extensive accumulation, which makes it difficult to establish a relationship between plasma levels and severe lactic acidosis [9]. For this reason, this drug should be suspected as a possible causative agent of acidosis in patients with relevant deterioration of renal function.

This case series shows that in patients with MALA, there are significant variations in the anion gap value, which is not related to the level of accumulated metformin, and therefore, it is doubtful whether calculating the anion gap could be used an approach for MALA diagnosis.

## Data Availability

Not applicable.

## Abbreviations

MALA: Metformin-associated lactic acidosis
MDRD: Modification of diet in renal disease

## References

[1] American Diabetes Association. 9. Pharmacologic approaches to glycemic treatment: standards of medical care in diabetes-2019. Diabetes Care. 2019;42(Suppl 1):S90–102. 10.2337/dc19-S009 30559235

[2] Taub ES, Hoffman RS, Manini AF. Incidence and risk factors for hyperlactatemia in ED patients with acute metformin overdose. Am J Emerg Med. 2019;37(12):2205–08. 10.1016/j.ajem.2019.03.033 PMC9881186 30967322

[3] Inzucchi SE, Lipska KJ, Mayo H, Bailey CJ, McGuire DK. Metformin in patients with type 2 diabetes and kidney disease: a systematic review. JAMA. 2014;312(24):2668–75. 10.1001/jama.2014.15298 PMC4427053 25536258

[4] Kajbaf F, Lalau J-D. Mortality rate in so-called “metformin-associated lactic acidosis”: a review of the data since the 1960s. Pharmacoepidemiol Drug Saf. 2014;23(11):1123–7. 10.1002/pds.3689 25079826

[5] Lalau J-D, Kajbaf F, Protti A, Christensen MM, De Broe ME, Wiernsperger N. Metformin-associated lactic acidosis (MALA): moving towards a new paradigm. Diabetes Obes Metab. 2017;19(11):1502–12. 10.1111/dom.12974 28417525

[6] Levey AS, Stevens LA, Schmid CH, Zhang YL, Castro AF 3rd, Feldman HI, *et al.* A new equation to estimate glomerular filtration rate. Ann Intern Med. 2009;150(9):604–12. 10.7326/0003-4819-150-9-200905050-00006 PMC2763564 19414839

[7] Graham GG, Punt J, Arora M, Day RO, Doogue MP, Duong JK. Clinical pharmacokinetics of metformin. Clin Pharmacokinet. 2011;50(2):81–98. 10.2165/11534750-000000000-00000 21241070

[8] Calello DP, Liu KD, Wiegand TJ, Roberts DM, Lavergne V, Gosselin S, *et al.* Extracorporeal treatment for metformin poisoning: systematic review and recommendations from the extracorporeal treatments in poisoning workgroup. Crit Care Med. 2015;43(8):1716–30. 10.1097/CCM.0000000000001002 25860205

[9] Kopec K, Kowalski MJ. Metformin-associated lactic acidosis (MALA): case files of the Einstein Medical Center medical toxicology fellowship. J Med Toxicol. 2013;9(1):61–6. 10.1007/s13181-012-0278-3 PMC3576503 23233435

[10] Kreisberg RA. Lactate homeostasis and lactic acidosis. Ann Intern Med. 1980;92(2 Pt 1):227–37. 10.7326/0003-4819-92-2-227 6766289

[11] Adams BD, Bonzani TA, Hunter CJ. The anion gap does not accurately screen for lactic acidosis in emergency department patients. Emerg Med J. 2006;23(3):179–82. 10.1136/emj.2005.026096 PMC2464445 16498152

[12] Kajbaf F, Lalau JD. The prognostic value of blood pH and lactate and metformin concentrations in severe metformin-associated lactic acidosis. BMC Pharmacol Toxicol. 2013;14:22. 10.1186/2050-6511-14-22 PMC3637618 23587368

